# Helix-Sense-Selective Polymerization of 3,5-bis(hydroxymethyl)phenylacetylene Rigidly Bearing Galvinoxyl Residues and Their Chiroptical Properties

**DOI:** 10.3390/polym11111877

**Published:** 2019-11-13

**Authors:** Zhichun Shi, Jianjun Wang, Masahiro Teraguchi, Toshiki Aoki, Takashi Kaneko

**Affiliations:** 1 College of Chemistry and Chemical Engineering, Qiqihar University, Wenhua Street 42, Qiqihar 161006, Heilongjiang, China; 2 Department of Chemistry and Chemical Engineering, Niigata University, Ikarashi 2-8050, Nishi-ku, Niigata 950-2181, Japan; teraguti@eng.niigata-u.ac.jp (M.T.); toshaoki@eng.niigata-u.ac.jp (T.A.); 3 Graduate School of Science and Technology, Niigata University, Ikarashi 2-8050, Nishi-ku, Niigata 950-2181, Japan; 4 College of Materials Science and Engineering, Qiqihar University, Wenhua Street 42, Qiqihar 161006, Heilongjiang, China; wangjianjun860505@163.com

**Keywords:** helix-sense-selective polymerization, conjugated polymer, poly(phenylacetylene), polyradical

## Abstract

Four kinds of newly synthesized achiral phenylacetylenes bearing a phenylhydrogalvinoxyl residue at 4-position were polymerized by using a chiral rhodium catalyst system, [Rh(nbd)B(C_6_H_5_)_4_] or [Rh(nbd)Cl]_2_ catalysts in the presence of chiral (*R*)-(+)- or (*S*)-(–)-1-phenylethylamine ((*R*)- or (*S*)-PEA) cocatalysts. Poly(*m*-HGDHPA) and poly(*m*-HGTHPA) in THF showed Cotton signals at the absorption regions of the main chain and hydrogalvinoxyl in the circular dichroism (CD) spectra. It indicated that excess of one-handed helical polyacetylene backbone was induced by helix-sense-selective polymerization (HSSP) under the asymmetric conditions despite the achiral monomer, and the hydrogalvinoxyl moieties were also arranged to form one-handed helical structure. However, there was no Cotton effect for poly(*p*-HGDHPA) and poly(*p*-HGTHPA) because the intramolecular hydrogen bonding did not act well to stabilize the helical conformation. The hydrogalvinoxyl units of poly(*m*-HGDHPA) and poly(*m*-HGTHPA) were converted to the corresponding galvinoxyl radicals after treatment with PbO_2_. In the CD spectra of the polyradicals, the Cotton effects decreased depending on their static stability of helical conformation, suggesting that reversal conformation of the polymer chain arose.

## 1. Introduction

Polymers with helical conformation possess chirality; i.e., the polymers predominantly folding into either a left- or right-handed helical conformation exhibit optical activity despite the absence of asymmetric carbons. Synthesis of helical polymers with a controlled helicity has attracted considerable attention as one of the fundamental factors for their wide variety of potential applications in materials science, such as chiral sensors, chiral catalysts, optical resolution, microelectronic devices, organic magnetic materials, and so on [1,2,3,4,5,6,7,8,9,10,11,12,13,14,15,16,17]. 

**Chart 1:**
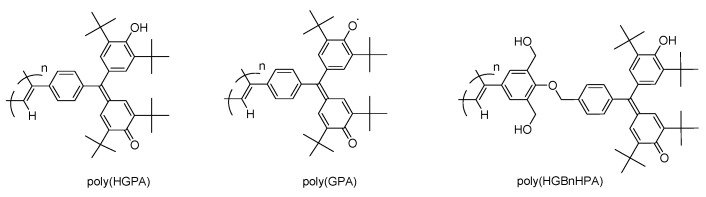
Poly(phenylacetylene)s bearing hydrogalvinoxyl residues.

Some of organic radicals are so stable as to remain from a few months to a few years in ambient atmosphere. [4-[[3,5-Bis(1,1-dimethylethyl)-4-oxo-2,5-cyclohexadien-1-ylidene]methyl]-2,6-bis(1,1- dimethylethyl)phenoxy] (abbreviated as galvinoxyl), one of stable free radicals, is known to exhibit interesting magnetic behavior [18]. Various π-conjugated polymers bearing galvinoxyls have been synthesized and their magnetic properties have been discussed in connection with their chemical structures and electronic states [19,20,21,22,23,24,25]. Some of the polyradicals had one-handed helical conformation and the relatively strong antiferromagnetic interaction of the polyradicals was caused by close packing between galvinoxyl radicals induced by the formation of the folded helical structure [26]. Optically active helical polyacetylenes have been investigated as functional polymers based on their chiral structure [27,28,29], and optically active helical poly(phenylacetylene)s bearing hydrogalvinoxyl residues (poly(HGPA)) were also synthesized by the authors (Niigata) (Chart 1) [30,31,32]. However, no Cotton effect was observed in the absorption region of the galvinoxyl radical chromophore for the corresponding polyradicals (poly(GPA)). On the other hand, the authors (Niigata) discovered helix-sense-selective polymerization (HSSP) of an achiral 3,5- bis(hydroxymethyl)-4-dodecyloxyphenylacetylene (DHPA) by using a chiral catalytic system in 2003 [6]. The one-handed helicity is stable and static in nonpolar solvents because it is maintained by intramolecular hydrogen bonds. The HSSP of the DHPA analogue bearing a hydrogalvinoxyl residue (HGBnHPA) also proceeded to give the corresponding polymer (poly(HGBnHPA)) whose one-handed helical backbone was statically stabilized by the intramolecular hydrogen bonds. However, the Cotton effect was not almost observed in the absorption region of the galvinoxyl moiety because the galvinoxyl moiety was linked by flexible benzyl linker [33,34]. The challenge of the optically active helical poly(phenylacetylene) that combine helical chirality and galvinoxyl residues linked by a rigid linker will be valuable for the development of optically active π-conjugated polymer materials.

Several 3,5-bis(hydroxymethyl)phenylacetylene monomers connected with rigid and linear π-conjugated substituents at the 4-position (3,5-bis(acetoxymethyl)-4- (4-dodecyloxyphenyl)phenylacetylene (DPHPA), 3,5-bis(hydroxymethyl)-4-(4’-dodecyloxy[1,1’-biphenyl]-4-yl)phenylacetylene (DBHPA), 3,5-bis(hydroxymethyl)-4-(4’-dodecyloxy-4-tolanyl)phenylacetylene (DTHPA), and 3,5-bis(hydroxymethyl)-4-[4’-[(4-dodecyloxyphenyl)ethynyl]-4-tolanyl]phenylacetylene (DPETHPA)) were synthesized via the Suzuki-Miyaura coupling using the corresponding precursor with triflate and their HSSP abilities were investigated [9,15,16]. However, there are no reports concerning the synthesis of 3,5-bis(hydroxymethyl)phenylacetylene bonded to galvinoxyl residues by a rigid linker.

**Scheme 1:**
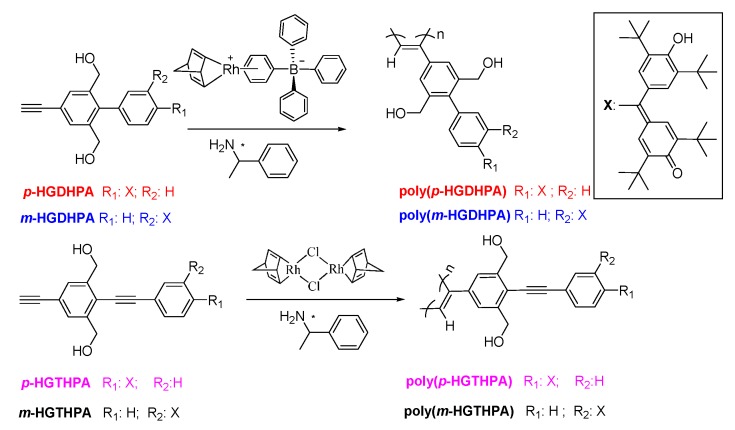
Polymerization of monomers having phenylhydrogalvinoxyl moiety under chiral rhodium catalyst system.

In this paper, we report synthesis of 3,5-bis(hydroxymethyl)phenylacetylenes rigidly bearing a hydrogalvinoxyl moiety (*p*- and *m*-HGDHPA, and *p*- and *m*- HGTHPA) and polymerization of the monomers under chiral rhodium catalyst system to result in inducing the excess of one-handed helical structure of the polyacetylene backbone and galvinoxyl radical chromophores for *m*-HGDHPA and *m*-HGTHPA (Scheme 1).

## 2. Experimental Section 

### 2.1. Materials

All the solvents used for monomer synthesis and polymerization were distilled before use. The polymerization initiators, [Rh(nbd)Cl]_2_ and [Rh(nbd)B(C_6_H_5_)_4_] (nbd=2,5 norbornadiene), purchased from Aldrich Chemical Co., Inc. (Milwaukee, WI, USA), was used as received. The silicon agents such as trimethylsilylacetylene were obtained from Shinetsu Chemical Co., Ltd. (*S*)-(−)- and (*R*)-(+)-1-Phenylethylamine were purchased from Tokyo Chemical Industry Co. (Tokyo, Japan). 2,6-Bis(acetoxymethyl)-4-(trimethylsilylethynyl)phenyl trifluoromethanesulfonate (**1**) was synthesized according to our previous report [15]. 4-(Methoxycarbonyl)phenylboronic acid (**2**) and 3-(methoxycarbonyl)phenylboronic acid (**3**) were purchased from Tokyo Chemical Industry Co. (Tokyo, Japan). 2,6-Di-*tert*-butyl-4-bromophenoxy)trimethylsilane (**4**) [35], methyl 4-ethynylbenzoate (**5**), and methyl 3-ethynylbenzoate (**6**) [36] were synthesized according to the literature method.

### 2.2. Monomers Synthesis 

#### 2.2.1. {2-[4-(Methoxycarbonyl)phenyl]-5-[(trimethylsilyl)ethynyl]-1,3-phenylene}bis(methylene) diacetate (**7**)

Compound **1** (0.933 g, 2.00 mmol), **2** (0.540 g, 3.00 mmol), Pd(OAc)_2_ (8.98 mg 0.0400 mmol), S-Phos (32.8 mg, 0.0800 mmol), and K_3_PO_4_ (1.27 g, 6.00 mmol) were dissolved in dioxane (15 mL) under nitrogen atmosphere. The solution was stirred at 100 °C for 12 h. The resulting solution was cooled to room temperature. The resulting mixture was extracted with ethyl acetate and then dried over anhydrous sodium sulfate. The solvent was removed using a rotary evaporator. The crude product was purified by silica-gel column chromatography (ethyl acetate: hexane = 1:3) to give **7** as colorless solid. R_f_ = 0.28; yield: 0.600 g (66.3%); ^1^H NMR (400 MHz, CDCl_3_), δ: 8.09 (d, *J* = 8.2 Hz, 2H, Ar-H), 7.55 (s, 2H, Ar-H), 7.27 (d, *J* = 9.4 Hz, 2H, Ar-H), 4.73 (s, 4H, -CH_2_-), 3.95 (s, 3H, Ar-COOCH_3_), 2.00 (s, 6H, -OCOCH_3_), 0.27(s, 9H, TMS); ^13^C NMR (100 MHz, CDCl_3_): δ: 170.35, 166.63, 141.52, 140.69, 134.45, 132.19, 129.88, 129.67, 129.26, 123.35, 103.86, 95.59, 63.63, 52.27, 20.84, -0.10.

#### 2.2.2. 4-[2,6-Bis(hydroxymethyl)-4-ethynylphenyl]phenylhydrogalvinoxyl (*p*-HGDHPA)

Compound **4** (2.48 g, 6.93 mmol) was dissolved in THF (18 mL) under nitrogen atmosphere. The solution was stirred at −78 °C for 10 min. After *n*-BuLi (4.22 mL, 6.93 mmol in hexane) was slowly added dropwise to the solution, the solution was stirred at −78 °C for 30 min. Then, the TMEDA (0.344 mL, 2.31 mmol) was added dropwise to the reaction solution, and the solution was stirred at −78 °C for 30 min. After **7** (0.448 g, 0.990 mmol) in THF (5 mL) was added dropwise to the reaction solution, and the solution was stirred at −78 °C for 80 min. After the solution was stirred at room temperature for 120 min, KOH (0.4 g, 7.1 mmol) in methanol (10 mL) was added to the reaction solution, and the solution was stirred at room temperature for 12 h. After 10% NH_4_Cl was added to the resulting solution, the mixture was extracted with ethyl acetate and then dried over anhydrous sodium sulfate. After the solvent was removed using a rotary evaporator, the crude product was purified by silica-gel column chromatography (chloroform: ethyl acetate = 7:1) to give *p*-HGDHPA as orange powder. R_f_ = 0.47; yield: 558 mg (85.6%); ^1^H NMR (400 MHz, CDCl_3_), δ: 7.70 (s, 2H, Ar–H), 7.37 (d, *J* = 8.3 Hz, 2H, Ar–H), 7.31 (d, *J* = 2.5 Hz, 1H, Ar-H(quinoid)), 7.26 (d, *J* = 8.1 Hz, 2H, Ar–H), 7.10 (d, *J* = 2.6 Hz, 1H, Ar-H(quinoid)), 7.07 (s, 2H, Ar–H), 5.54 (s, 1H, Ar–OH), 4.42 (d, *J* = 5.8 Hz, 4H, –CH_2_–), 3.14 (s, 1H, -C≡C–H), 1.54 (t, *J* = 5.8 Hz, 2H, OH), 1.43 (s, 18H, *t*-Bu), 1.31 (s, 9H, *t*-Bu), 1.25 (s, 9H, *t*-Bu); ^13^C NMR (100 MHz, CDCl_3_), δ: 186.08, 156.93, 155.61, 147.08, 146.67, 141.17, 139.20, 139.01, 137.89, 135.36, 132.37, 132.34, 132.32, 131.49, 130.23, 129.99, 128.94, 128.37, 122.21, 82.25, 77.65, 62.67, 35.35, 35.24, 34.40, 30.23, 29.67, 29.38; IR (KBr, pellet): 3601 (O-H), 3375 (O-H), 3305 (≡C–H) cm^−1^.

#### 2.2.3. {2-[3-(Methoxycarbonyl)phenyl]-5-[(trimethylsilyl)ethynyl]-1,3-phenylene}bis(methylene)diacetate (8)

Compound **1** (1.40 g, 3.00 mmol), **3** (810 mg, 4.50 mmol), Pd(OAc)_2_ (13.5 mg 0.0600 mmol), S-Phos (49.3 mg, 0.120 mmol), and K_3_PO_4_ (1.91 g, 9.00 mmol) were dissolved in dioxane (15 mL) under nitrogen atmosphere. The solution was stirred at 100 °C for 12 h. The resulting solution was cooled to room temperature. The resulting mixture was extracted with ethyl acetate and then dried over anhydrous sodium sulfate. The solvent was removed using a rotary evaporator. The crude product was purified by silica-gel column chromatography (ethyl acetate: hexane = 2:7) to give **8** as yellow viscous liquid. R_f_ = 0.30; yield: 1.00 g (73.7%);^1^H NMR (700 MHz, CDCl_3_), δ: 8.07 (dt, *J* = 7.8, 1.4 Hz, 1H, Ar–H), 7.90 (t, *J* = 1.8 Hz, 1H, Ph–H), 7.56 (s, 2H, Ar–H), 7.51 (t, *J* = 7.7 Hz, 1H, Ar–H), 7.39 (dt, *J* = 7.6, 1.3 Hz, 1H, Ar–H), 4.73 (s, 4H, –CH_2_–), 3.91 (s, 3H, Ar–COOCH_3_), 2.02 (s, 6H, –CH_2_–OCOCH_3_), 0.28 (s, 9H, –Si–(CH_3_)_3_); ^13^C NMR (CDCl_3_), δ: 170.33, 166.54, 140.75, 136.81, 134.67, 133.44, 132.29, 130.34, 130. 26, 129.24, 128.52, 123.23, 103.88, 95.46, 63.71, 52.18, 20.73, -0.15, IR (KBr, pellet): 2143 (C≡C), 1740, 1720 (C=O) cm^−1^.

#### 2.2.4. 3-[2,6-Bis(hydroxymethyl)-4-ethynylphenyl]phenylhydrogalvinoxyl (*m*-HGDHPA)

Compound **4** (3.53 g, 9.87 mmol) was dissolved in THF (30 mL) under nitrogen atmosphere. The solution was stirred at −78 °C for 10 min. After n-BuLi (7.53 mL, 11.9 mmol in hexane) was slowly added dropwise to the solution, the solution was stirred at −78 °C for 30 min. Then, the TMEDA (0.490 mL, 3.29 mmol) was added dropwise to the reaction solution, and the solution was stirred at −78 °C for 30 min. After, **8** (0.640 g, 1.41 mmol) in THF (7 mL) was added dropwise to the reaction solution, and the solution was stirred at −78 °C for 80 min. After the solution was stirred at room temperature for 120 min, KOH (0.8 g, 14 mmol) in methanol (10 mL) was added to the reaction solution, and the solution was stirred at room temperature for 12 h. After 5% NH_4_Cl was added to the resulting solution, the mixture was extracted with ethyl acetate and then dried over anhydrous sodium sulfate. After the solvent was removed using a rotary evaporator, the crude product was purified by silica-gel column chromatography (chloroform: ethyl acetate = 7:1) to give *m*-HGDHPA as orange powder. R_f_ = 0.47; yield: 570 mg (61.4%); ^1^H NMR (400 MHz, CDCl_3_), δ: 7.63(s, 2H, Ar–H), 7.53 (t, *J* = 7.7 Hz, 1H, Ar–H), 7.38 (dt, *J* = 8.0 Hz, *J* = 1.4 Hz, 1H, Ar–H), 7.27 (dt, *J* = 9.0 Hz, *J* = 1.4 Hz, 1H, Ar–H), 7.26 (d, *J* = 2.6 Hz, 1H, Ar-H(quinoid)), 7.10 (d, *J* = 2.6 Hz, 1H, Ar–H(quinoid)), 7.02 (t, *J* = 1.7 Hz, 1H, Ar–H), 7.01 (s, 2H, Ar–H), 5.51 (s, 1H, Ar–OH), 4.37 (dd, *J* = 13, 6.1 Hz, 2H, –CH_2_–), 4.36 (dd, *J* = 13, 6.0 Hz, 2H, –CH_2_–), 3.09 (s, 1H, –C≡C–H), 1.45 (t, *J* = 6.0 Hz, 2H, –OH), 1.40 (s, 18H, *t*-Bu), 1.28 (s, 9H, *t*-Bu), 1.24 (s, 9H, *t*-Bu); ^13^C NMR (100 MHz, CDCl_3_), δ: 186.10, 156.82, 155.56, 147.14, 146.93, 142.10, 139.23, 138.91, 136.99, 135.44, 132.34, 131.99, 131.94, 131.76, 131.59, 130.20, 129.75, 129.40, 128.96, 128.28, 122.24, 83.18, 77.66, 62.69, 35.32, 35.23, 34.39, 30.20, 29.65, 29.48; IR (KBr, pellet): 3623, 3583 (O–H), 3490 (O–H), 3291 (≡C–H) cm^−1^.

#### 2.2.5. {2-{[4-(Methoxycarbonyl)phenyl]ethynyl}-5-[(trimethylsilyl)ethynyl]-1,3-phenylene}bis(methylene) diacetate (**9**)

Compound **5** (758 mg, 4.73 mmol) was added to a solution of **1** (2.0 g, 4.3 mmol), copper(I)iodide (49 mg 0.258 mmol), triphenylphosphine (45 mg, 0.172 mmol), and bis(triphenylphosphine)palladium(II) chloride (151 mg, 0.215 mmol) in triethylamine (125 mL) and the solution was refluxed for 24 h. The formed salt was removed by filtration and the solvent was concentrated to give a viscous liquid, which was purified by silica-gel column chromatography (hexane: ethyl acetate = 3:1) to give **9** as a yellow solid. R_f_ = 0.29; yield: 0.23 g (11%); ^1^H NMR (400 MHz, CDCl_3_), δ: 8.03 (d, *J* = 8.6 Hz, 2H, Ar-H), 7.58 (d, *J* = 8.6 Hz, 2H, Ar–H), 7.50 (s, 2H, Ar–H), 5.33 (s, 4H, –CH_2_–), 3.93 (s, 3H, Ar–COOCH_3_), 2.14 (s, 6H, –OCOCH_3_), 0.26 (s, 9H, –Si(CH_3_)_3_); ^13^C NMR (100 MHz, CDCl_3_), δ: 170.65, 166.37, 138.34, 131.49, 131.28, 130.17, 129.65, 126.96, 123.69, 121.12, 103.91, 100.26, 97.39, 86.09, 64.11, 52.25, 20.89, −0.17.

#### 2.2.6. 4-{[2,6-Bis(hydroxymethyl)-4-ethynylphenyl]ethynyl}phenylhydrogalvinoxyl (*p*-HGTHPA)

Compound **4** (1.7 g, 4.7 mmol) was dissolved in THF (20 mL) under nitrogen atmosphere, and the solution was stirred at −78 °C for 10 min. After, *n*-BuLi (3.1 mL, 4.7 mmol in hexane) was slowly added dropwise to the solution, the solution was stirred at −78 °C for 30 min. Then, the TMEDA (0.3 mL, 2 mmol) was added dropwise to the reaction solution, and the solution was stirred at −78 °C for 30 min. After, **9** (0.43 g, 0.90 mmol) in THF (5 mL) was added dropwise to the reaction solution, and the solution was stirred at −78 °C for 80 min. After the solution was stirred at room temperature for 120 min, KOH (0.36 g, 6.3 mmol) in methanol (10 mL) was added to the reaction solution, and the solution was stirred at room temperature for 12 h. After 5% NH_4_Cl (50 mL) was added to the resulting solution, the mixture was extracted with ethyl acetate and then dried over anhydrous magnesium sulfate. After the solvent was removed using a rotary evaporator, the crude product was purified by silica-gel column chromatography (hexane: ethyl acetate = 3:1) to give *p*-HGTHPA as an orange powder. R_f_ = 0.17; yield: 0.22 g (35%). ^1^H NMR (400 MHz, CDCl_3_), δ: 7.60 (s, 2H, Ar–H), 7.56 (d, *J* = 8.4 Hz, 2H, Ar–H), 7.30 (d, *J* = 8.4 Hz, 2H, Ar–H), 7.24 (d, *J* = 2.5 Hz, 1H, Ar–H(quinoid)), 7.11 (d, *J* = 2.5 Hz, 1H, Ar-H (quinoid)), 7.02 (s, 2H, Ar-H), 5.54 (s, 1H, Ar–OH), 4.95 (s, 4H, –CH_2_–), 3.19 (s, 1H, –C≡C–H), 1.42 (s, 18H, *t*-Bu), 1.28 (s, 9H, *t*-Bu), 1.26 (s, 9H, *t*-Bu); ^13^C NMR (100 MHz, CDCl_3_), δ: 186.08, 156.59, 155.61, 147.08, 147.00, 143.16, 141.96, 135.83, 135.44, 132.55, 132.02, 131.44, 130.85, 130.02, 129.66, 129.32, 123.01, 122.71, 119.24, 100.65, 85.35, 83.19, 79.23, 63.47, 35.31, 35.27, 34.40, 30.28, 29.65, 29.56; IR (KBr, pellet): 3606 (O–H), 3312 (≡C–H) cm^−1^.

#### 2.2.7. {2-{[3-(Methoxycarbonyl)phenyl]ethynyl}-5-[(trimethylsilyl)ethynyl]-1,3-phenylene}bis(methylene) diacetate (**10**)

Compound **6** (758 mg, 4.73 mmol) was added to a solution of **1** (2.0 g, 4.3 mmol), copper(I)iodide (49 mg 0.258 mmol), triphenylphosphine (45 mg, 0.172 mmol), and bis(triphenylphosphine)palladium(II) chloride (151 mg, 0.215 mmol) in triethylamine (125 mL) and the solution was refluxed for 24 h. The formed salt was removed by filtration, and the solvent was concentrated to give a viscous liquid, which was purified by silica-gel column chromatography (hexane: ethyl acetate = 3:1) to give **10** as a yellow solid. R_f_ = 0.27; yield: 0.88 g (43%); ^1^H NMR (700 MHz, CDCl_3_), δ: 8.17 (t, *J* = 1.4 Hz, 1H, Ar–H), 8.03 (dt, *J* = 7.8 Hz, 1.4 Hz, 1H, Ar–H), 7.70 (dt, *J* = 7.6, 1.4 Hz, 1H, Ar–H), 7.50 (s, 2H, Ar–H), 7.46 (t, *J* = 7.8 Hz, 1H, Ar–H), 5.33 (s, 4H, –CH_2_–), 3.94 (s, 3H, Ar–COOCH_3_), 2.15 (s, 6H, –OCOCH_3_), 0.26 (s, 9H, –Si–(CH_3_)_3_); ^13^CNMR (175 MHz, CDCl_3_), δ: 170.66, 166.20, 138.23, 135.69, 132.54, 131.15, 130.61, 129.93, 128.70, 123.42, 122.83, 121.20, 103.94, 100.06, 97.17, 84.15, 64.11, 52.34, 20.91, −0.17.

#### 2.2.8. 3-{[2,6-Bis(hydroxymethyl)-4-ethynylphenyl]ethynyl}phenylhydrogalvinoxyl (*m*-HGTHPA)

Compound **4** (1.1 g, 3.0 mmol) was dissolved in THF (20 mL) under nitrogen atmosphere, and the solution was stirred at −78 °C for 10 min. After *n*-BuLi (1.94 mL, 3.0 mmol in hexane) was slowly added dropwise to the solution, the solution was stirred at −78 °C for 30 min. Then, the TMEDA (0.15 mL, 1 mmol) was added dropwise to the reaction solution, and the solution was stirred at −78 °C for 30 min. After **10** (0.28 g, 0.58 mmol) in THF (5 mL) was added dropwise to the reaction solution, and the solution was stirred at −78 °C for 80 min. After the solution was stirred at room temperature for 120 min, KOH (0.23 g, 4.06 mmol) in methanol (10 mL) was added to the reaction solution, and the solution was stirred at room temperature for 12 h. After 5% NH_4_Cl (50 mL) was added to the resulting solution, the mixture was extracted with ethyl acetate and then dried over anhydrous magnesium sulfate. After the solvent was removed using a rotary evaporator, the crude product was purified by silica-gel column chromatography (hexane: ethyl acetate = 3:1) to give *m*-HGTHPA as an orange powder. R_f_ = 0.19; yield: 0.16 g (42%); ^1^H NMR (700 MHz, CDCl_3_), δ: 7.58 (dt, *J* = 7.8, 1.3 Hz, 1H, Ar-H), 7.58 (s, 2H, Ar–H), 7.45 (t, *J* = 1.4 Hz, 1H, Ar–H), 7.42 (t, *J* = 7.8 Hz, 1H, Ar–H), 7.27 (dt, *J* = 7.8, 1.3 Hz, 1H, Ar–H), 7.26 (d, *J* = 2.5 Hz, 1H, Ar–H (quinoid)), 7.08 (d, *J* = 2.5 Hz, 1H, Ar–H(quinoid)), 7.03 (s, 2H, Ar–H), 5.54 (s, 1H, Ar–OH), 4.89 (s, 4H, –CH_2_–), 3.17 (s, 1H, –C≡C–H), 2.07 (s, 2H, –OH), 1.41 (s, 18H, t-Bu), 1.29 (s, 9H, t-Bu), 1.24 (s, 9H, t-Bu).

### 2.3. Polymerization

A solution of [Rh(nbd)B(C_6_H_5_)_4_] or [Rh(nbd)Cl]_2_ and (*R*)- or (*S*)-phenylethylamine (PEA) in THF or toluene ([PEA]/[Cat.] = 400, or 800) was added to a solution of monomer (*p*-HGDHPA, *m*-HGDHPA, *p*-HGTHPA and *m*-HGTHPA) in THF or toluene ([M] = 0.1 or 0.2 M, [M]/[Cat.] = 100). The reaction solution was stirred at room temperature for 24 h or 96 h. The crude polymer was purified by reprecipitation of the reaction solution into a large amount of mixed solvent (diethyl ether: hexane = 1:2) and dried in vacuo to give an orange solid. The polymerization data for these resultant polymers are summarized in Table 1. 

### 2.4. Oxidation

The PbO_2_ (50 eq.) was added to a solution of the polymers in anhydrous THF under nitrogen in a glovebox. The solution was stirred for 1 h, then PbO_2_ was removed by membrane filtration to give the corresponding polyradicals solution, which was used for spectroscopic measurements.

### 2.5. Measurements

NMR (^1^H, ^13^C) spectra were recorded on a Varian FT-NMR 400MR (400 MHz) or System 700 (700 MHz) spectrometer (Varian, Palo Alto, CA, USA). Average molecular weights (*M*_n_ and *M*_w_) were determined by gel-permeation chromatography on two Shodex columns (KF-807L, eluent THF, Tokyo, Japan) in a liquid chromatograph device (JASCO, Tokyo, Japan) equipped with a UV detector (UV-2070) and calibrated using polystyrene standards. CD/UV-vis spectra were recorded on a J-720WI spectropolarimeter (JASCO, Tokyo, Japan) with a Peltier temperature controller. 

## 3. Results and Discussion

**Scheme 2:**
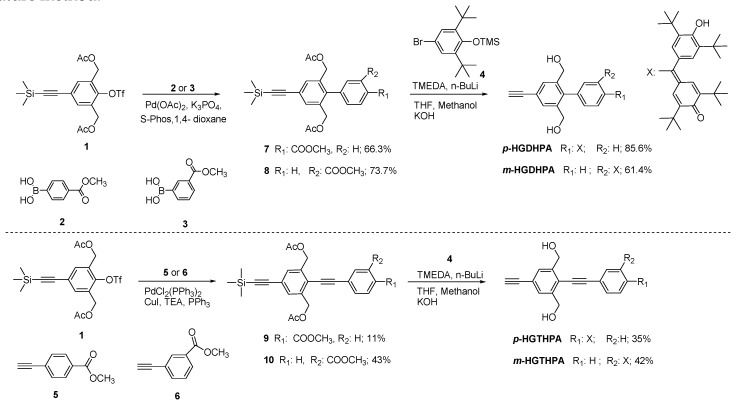
The synthetic route of monomers.

The monomers were synthesized from the corresponding precursor **1** with triflate as the leaving group via the palladium complex-catalyzed coupling reaction, as shown in Scheme 2. Suzuki-Miyaura coupling reaction of **1** which had two bulky acetoxymethyl groups at the *o*-positions was improved by using S-Phos as ligands compared with the previous work [9]. On the other hand, Sonogashira coupling reaction of **1** and phenylacetylenes **5** and **6** gave the corresponding products in 10−40% yield. Four new monomers (*p*-HGDHPA, *m*-HGDHPA, *p*-HGTHPA, and *m*-HGTHPA) were obtained by coupling reaction of the corresponding methyl benzoates **7**, **8**, **9**, and **10** with (2,6-di-tert-butyl-4-lithiophenoxy)trimethylsilane, which addition of six times equilibrium gave the products in 35−85% yield. 

We have already succeeded in the HSSP of various 4-substituted 3,5-bis(hydroxymethyl)phenylacetylenes [6,9,10,11,13,14,15,16]. Then, we explore the HSSP of new achiral monomers rigidly bearing a hydrogalvinoxyl moiety. Polymerization of *p*-HGDHPA, *m*-HGDHPA, *p*-HGTHPA, and *m*-HGTHPA were carried out in the presence of the Rh complex catalysts and chiral PEA as shown in Table 1. Addition of copper(I) iodide (CuI) into the catalytic system improved the polymer yield (Table 1 no. 3 and 4), which was consistent with the previously reported poly(HGBnHPA) [33]. Solubility of the obtained polymers was maintained due to the peripheral hydrogalvinoxyl moiety. The polymers dissolved well in common organic solvents, such as chloroform, tetrahydrofuran (THF), and toluene.

**Figure 1:**
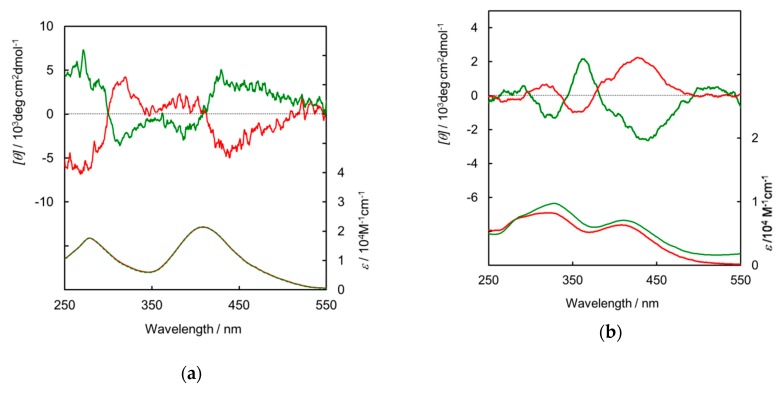
CD and UV-vis spectra of (**a**) poly(*m*-HGDHPA) (red line: no.3 in Table 1; green line: no.4 in Table 1) and (**b**) poly(*m*-HGTHPA) (red line: no.6 in Table 1; green line: no.7 in Table 1) at 20 °C in THF.

The chiroptical properties of the isolated polymers were initially investigated by circular dichroism (CD) in THF at 20 °C. Two split-type induced CD signals at 300 and 420 nm and broad signals at 450 nm were observed for the THF solution of poly(*m*-HGDHPA) and poly(*m*-HGTHPA) as shown in Figure 1. The CD spectra of the polymers obtained by polymerization using (*R*)-PEA and (*S*)-PEA as co-catalyst are mirror images of each other, indicating their enantiomeric conformations. The Cotton effect around 300–310 nm was often observed for poly[3,5-bis(hydroxymethyl)phenylacetylene]s [6] and attributed to phenylene chromophore attached to polyacetylene backbone with one-handed helical structure. The absorption bands at 420 nm and 450 nm are assigned to the hydrogalvinoxyl and polyacetylene backbone chromophore, respectively. The exciton coupled CD signal at 420 nm and the broad CD signal around visible region were observed for poly(HGPA) synthesized by HSSP [30,31,32]. The CD spectra of poly(*m*-HGDHPA) and poly(*m*-HGTHPA) can be explained by combination of the poly[3,5-bis(hydroxymethyl)phenylacetylene]s and the poly(HGPA), i.e., HSSP of *m*-HGDHPA and *m*-HGTHPA successfully proceeded and the excess of one-handed helical structure was not only induced in the polyacetylene backbone but also the hydrogalvinoxyl moiety. Although the sign of the CD signals at 300 nm agreed for poly(*m*-HGDHPA) and poly(*m*-HGTHPA) synthesized using the same enantiomeric PEA, the exciton coupled CD signals around 400 nm and the broad CD signals around visible region exhibited the opposite sign of each other. This indicates that the HSSP of *m*-HGDHPA and *m*-HGTHPA gave the opposite helix sense of each other, and helix-sense-selectivity of this chiral catalyst system was affected by the monomer structure. On the other hand, no optical activity was observed for poly(*p*-HGDHPA) and poly(*p*-HGTHPA), which were prepared under the same polymerization conditions as poly(*m*-HGDHPA) and poly(*m*-HGTHPA). These results did not conflict with our previous report [15] in which the stability of one-handed helical structure depended on the length of side group for poly[3,5-bis(hydroxymethyl)phenylacetylene]s connected with rigid and linear π-conjugated substituents at the 4-position.

**Figure 2:**
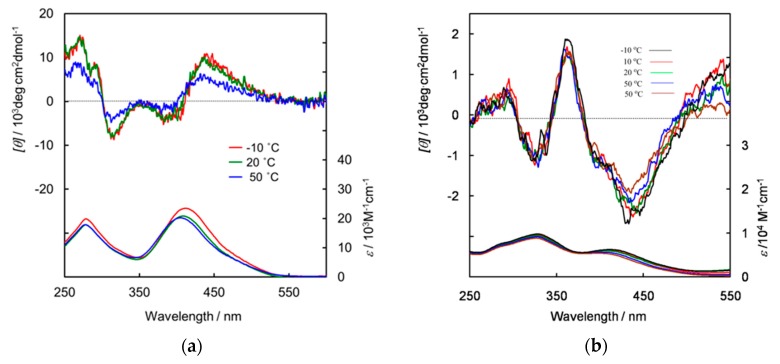
CD and UV-vis absorption spectra of (**a**) poly(*m*-HGDHPA) (no.5 in Table 1) and (**b**) poly(*m*-HGTHPA) (no.7 in Table 1) in THF at −10 °C–50 °C.

The stability of helical structure for poly(*m*-HGDHPA) and poly(*m*-HGTHPA) was clarified by temperature- and polar solvent addition-dependence of the CD spectra (Figure 2, Figures S1 and S2). We have already reported that the helical structure of poly[3,5-bis(hydroxymethyl)phenylacetylene]s was stabilized by intramolecular hydrogen bonds of the hydroxymethyl groups, and the CD intensity of poly(HGBnHPA) was nearly constant, even when the solution was heated to 60 °C [34]. The CD intensity of poly(m-HGDHPA) at 50 °C decreased to half from that at 20 °C, while the CD intensity of poly(m-HGTHPA) was nearly constant over the temperature range from −10 °C to 50 °C. The poly(*m*-HGDHPA) has the bis(hydroxymethyl)-substituted biphenyl moiety whose bond would be highly twisted by the steric hindrance induced by the ortho-substituent, and the highly twisted structure would enhance the steric repulsion between adjacent side groups to result in weakening the static stability of the helical conformation. Since the Cotton effect was vanished by adding DMSO as polar solvent in the THF solution of poly (*m*-HGDHPA) and poly(*m*-HGTHPA), it is proved that the helical conformation was stabilized by the hydrogen bonding (Figures S1 and S2). The weak hydrogen bonding of poly(*m*-HGDHPA) was confirmed from a broad peak at 3480 cm^−1^ attributed to ν_O-H_ of hydrogen bonding hydroxymethyl groups for IR spectra of poly(*m*-HGDHPA) in THF (Figure S3) compared with that of poly(DHPA) (ν_O-H_ = 3300 cm^−1^) [6], which would contribute to decreasing the static stability of the helical conformation.

**Scheme 3:**
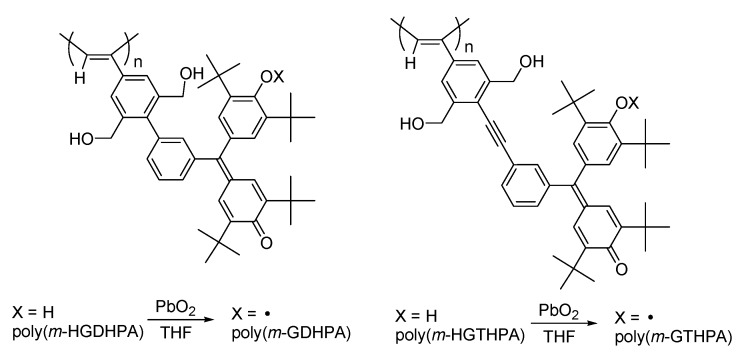
The synthetic route of poly(*m*-G**T**HPA) and poly(*m*-G**D**HPA).

**Figure 3:**
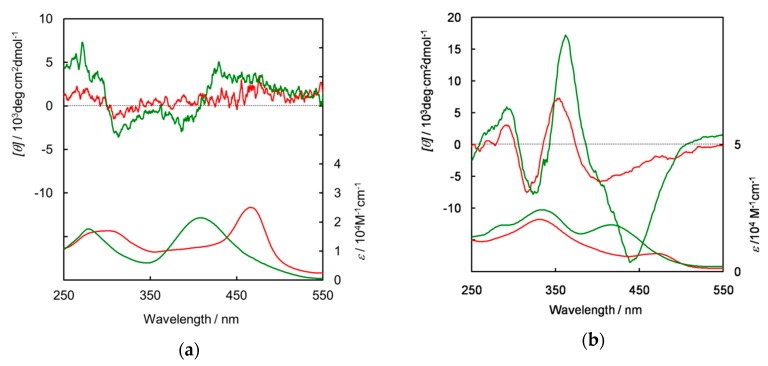
CD and UV-vis absorption spectra of (**a**) poly(*m*-HGDHPA) (no.4 in Table 1) (green line) and poly(*m*-GDHPA) (red line), and (**b**) poly(*m*-HGTHPA) (no.8 in Table 1) (green line) and poly(*m*-GTHPA) (red line) at 20 °C in THF.

The polyradicals poly(*m*-GDHPA) and poly(*m*-GTHPA) were obtained by oxidizing the corresponding polymers poly(*m*-HGDHPA) and poly(*m*-HGTHPA) in the treatment of the polymer solution on degassed THF with fresh lead dioxide under an oxygen-free atmosphere, respectively (Scheme 3). The UV-vis absorption spectra of the polyradicals exhibit decrease of the absorption maximum at 420 nm due to the hydrogalvinoxyl chromophore and appearance of a new absorption peak due to the galvinoxyl radical chromophore at 470 nm (Figure 3). The CD intensity of poly(*m*-GDHPA) was reduced by oxidation process of poly(*m*-HGDHPA) because the helical conformation of poly(*m*-HGDHPA) was not static enough to maintain its own helicity, and the contribution of quinone methide structure was reduced by the oxidation of hydrogalvinoxyl to lead to enhancement of the mobility of galvinoxyl structure. On the other hand, the CD intensity of poly(*m*-GTHPA) around 300 nm was nearly constant through the oxidation of poly(*m*-HGTHPA), indicating maintenance of the excess of the one-handed helical conformation. The CD intensity of poly(*m*-GTHPA) around 350–500 nm decreased through the oxidation of poly(*m*-HGTHPA), but weak split-type induced CD signal at 470 nm overlapped on the broad signal. This behavior is probably explained as follows. The Cotton effects around 350–500 nm included exciton couplings between (hydro)galvinoxyl chromophores and between (hydro)galvinoxyl chromophore and diphenylacetylene chromophore. Incomplete transformation to galvinoxyl radical state reduced the exciton coupling between the same galvinoxyl chromophores, and bathochromic shift from the hydrogalvinoxyl to the galvinoxyl radical weakened the exciton coupling between the galvinoxyl chromophore and the diphenylacetylene chromophore.

## 4. Conclusions

Helix-sense-selective polymerization of two kinds of achiral phenylacetylenes, *m*-HGDHPA and *m*-HGTHPA, was achieved by using a chiral rhodium catalyst system, [Rh(nbd)Cl]_2_ or Rh(nbd)B(C_6_H_5_)_4_ as an achiral catalyst and (*R*)- or (*S*)-PEA as a chiral cocatalyst. The chemical oxidation of poly(*m*-HGTHPA) yielded the corresponding optically active helical polyradical poly(*m*-GTHPA) and the excess of one-handed helical structure was not only induced in the polyacetylene backbone but also the galvinoxyl moiety. This is the first example for the polyradical prepared by HSSP of achiral phenylacetylenes bearing galvinoxyl residues.

## Figures and Tables

**Table 1:** Polymerization of *p*-HGDHPA, *m*-HGDHPA, *p*-HGTHPA, and *m*-HGTHPA using Rh complex catalysts in the presence of chiral PEA.

Run	Monomer	Solvent	PEA	Yield ^e^ (%)	*M*_w_^f^(10^5^)	*M*_w_/*M*_n_
1 ^a^	*p*-HGDHPA	THF	(*R*)-PEA	30	- ^g^	- ^g^
2 ^a^		THF	(*S*)-PEA	23	- ^g^	- ^g^
3 ^a^	*m*-HGDHPA	THF	(*R*)-PEA	74	3.4	3.0
4 ^a^		THF	(*S*)-PEA	88	5.3	2.9
5 ^b^		THF	(*S*)-PEA	17	24	7.8
6 ^c^	*m*-HGTHPA	THF	(*R*)-PEA	42	- ^g^	- ^g^
7 ^c^		THF	(*S*)-PEA	10	10	10
8 ^d^		THF	(*S*)-PEA	23	1.0	2.9
9 ^c^	*p*-HGTHPA	toluene	(*R*)-PEA	46	0.14	2.1
10 ^c^		toluene	(*S*)-PEA	27	0.13	3.1

^a^ [M] = 0.1 M, [M]/[Rh(nbd)B(C_6_H_5_)_4_] = 100, [PEA]/[Cat.] = 400, ([CuI]/[Cat.] = 1 for no. 3 and 4), 25 °C, 3 h; ^b^ [M] = 0.1 M, [M]/[[Rh(nbd)Cl]_2_] = 100, [PEA]/[Cat.] = 400, 25 °C, 3 h; ^c^ [M] = 0.1 M, [M]/[[Rh(nbd)Cl]_2_] = 100, [PEA]/[Cat.] = 800, 25 °C, 24 h; ^d^ [M] = 0.2 M, [M]/[[Rh(nbd)Cl]_2_] = 100, [PEA]/[Cat.] = 400, 25 °C, 96 h; ^e^ diethylether: hexane = 1:2 insoluble part; ^f^ Determine by GPC (eluent: THF, polystyrene standard); ^g^ Too broad GPC profile to determine quantitative average molecular weight.

## Supplementary Materials

The following are available online at https://www.mdpi.com/2073-4360/11/11/1877/s1. Figure S1: CD and UV-vis absorption spectra of poly(*m*-HGDHPA) in THF/DMSO with various compositions. Figure S2: CD and UV-vis absorption spectra of poly(*m*-HGTHPA) at 20 °C in THF/DMSO. Figure S3: IR spectra of poly(*m*-HGDHPA) in THF/DMSO with various compositions. Figure S4: UV-vis absorption spectra of poly(*m*-HGTHPA) (red line: oxidation of poly(*m*-HGTHPA), green line: poly(*m*-HGTHPA)) at 20 °C in THF. Figure S5: ^1^H NMR (CDCl_3_, 400 MHz) spectrum of *p*-HGDHPA. Figure S6: ^1^H NMR (CDCl_3_, 400 MHz) spectrum of *m*-HGDHPA. Figure S7: ^1^H NMR (CDCl_3_, 400 MHz) spectrum of *p*-HGTHPA. Figure S8: ^1^H NMR (CDCl_3_, 700 MHz) spectrum of *p*-HGTHPA. Figure S9: ^1^H NMR (CDCl_3_, 400 MHz) spectrum of poly(*m*-HGDHPA). Figure S10: GPC profile of poly(*p*-HGDHPA) in Table 1 no. 1. Figure S11: GPC profile of poly(*p*-HGDHPA) in Table 1 no. 2. Figure S12: GPC profile of poly(*m*-HGDHPA) in Table 1 no. 3. Figure S13: GPC profile of poly(*m*-HGDHPA) in Table 1 no. 4. Figure S14: GPC profile of poly(*m*-HGDHPA) in Table 1 no. 5. Figure S15: GPC profile of poly(*m*-HGTHPA) in Table 1 no. 6. Figure S16: GPC profile of poly(*m*-HGTHPA) in Table 1 no. 7.

## References

[1] Akagi K. (2009). Helical polyacetylene: Asymmetric polymerization in a chiral liquid-crystal field. Chem. Rev..

[2] Yashima E., Maeda K., Iida H., Furusho Y., Nagai K. (2009). Helical polymers: Synthesis, structures, and functions. Chem. Rev..

[3] Freire F., Quiñoá E., Riguera R. (2016). Supramolecular Assemblies from Poly(Phenylacetylene)S. Chem. Rev..

[4] Yashima E., Ousaka N., Taura D., Shimomura K., Ikai T., Maeda K. (2016). Supramolecular helical systems: Helical assemblies of small molecules, foldamers, and polymers with chiral amplification and their functions. Chem. Rev..

[5] Liu L.J., Zang Y., Jia H.G., Aoki T., Kaneko T., Hadano S., Teraguchi M., Miyata M., Zhang G., Namikoshi T. (2017). Helix-sense-selective polymerization of achiral phenylacetylenes and unique properties of the resulting cis-cisoidal polymers. Polym. Rev..

[6] Aoki T., Kaneko T., Maruyama N., Sumi A., Takahashi M., Sato T., Teraguchi M. (2003). Helix-sense-selective polymerization of phenylacetylene having two hydroxy groups using a chiral catalytic system. J. Am. Chem. Soc..

[7] Akagi K., Piao G., Kaneko S., Sakamaki K., Shirakawa H., Kyotani M. (1998). Helical polyacetylene synthesized with a chiral nematic reaction field. Science.

[8] Yashima E., Maeda K., Okamoto Y. (1999). Memory of macromolecular helicity assisted by interaction with achiral small molecules. Nature.

[9] Shi Z.C., Murayama Y., Kaneko T., Teraguchi M., Aoki T. (2013). Helix-sense-selective polymerization of 3,5-bis(hydroxymethyl)phenylacetylene connected with a rigid and π-conjugated substituent. Chem. Lett..

[10] Teraguchi M., Nahata N., Nishimura T., Aoki T., Kaneko T. (2019). Helix-sense-selective polymerization of phenylacetylenes having a porphyrin and a zinc-porphyrin group: One-handed helical arrangement of porphyrin pendants. Polymers.

[11] Wang Q.Y., Jia H.G., Shi Y.Q., Ma L.Q., Yang G.X., Wang Y.Z., Xu S.P., Wang J.J., Zang Y., Aoki T. (2018). Rh (L-alaninate) (1,5-Cyclooctadiene) catalyzed helix-sense-selective polymerizations of achiral phenylacetylenes. Polymers.

[12] Liu L.J., Zhang G., Aoki T., Wang Y., Kaneko T., Teraguchi M., Zhang C., Dong H. (2016). Synthesis of one-handed helical block copoly(substituted acetylene)s consisting of dynamic cis-transoidal and static cis-cisoidal block: Chiral teleinduction in helix-sense-selective polymerization using a chiral living polymer as an initiator. Acs Macro Lett..

[13] Hadano S., Kishimoto T., Hattori T., Tanioka D., Teraguchi M., Aoki T., Kaneko T., Namikoshi T., Marwanta E. (2009). Helix-sense-selective polymerization of achiral bis(hydroxymethyl)phenylacetylenes bearing alkyl groups of different lengths. Macromol. Chem. Phys..

[14] Liu L.J., Zang Y., Hadano S., Aoki T., Teraguchi M., Kaneko T., Namikoshi T. (2010). New achiral phenylacetylene monomers having an oligosiloxanyl group most suitable for helix-sense-selective polymerization and for obtaining good optical resolution membrane materials. Macromolecules.

[15] Shi Z.C., Teraguchi M., Aoki T., Kaneko T. (2015). Helical conformation stability of poly 3,5-bis (hydroxymethyl) phenylacetylenes depending on the length of their rigid and linear π-conjugated side groups. Chem. Lett..

[16] Shi Z.C., Kwak G., Jin Y.J., Teraguchi M., Aoki T., Kaneko T. (2018). Solvent-tuned dual emission of a helical poly [3,5-bis(hydroxymethyl) phenylacetylene] connected with a π-conjugated chromophore. Polym. J..

[17] Shen J., Ikeda N., Bi W., Satoh K., Kamigaito M., Okamoto Y. (2019). Helix-sense-selective copolymerization of triphenylmethyl methacrylate with chiral 2-isopropenyl-4-phenyl-2-oxazoline. J. Polym. Sci. Part A: Polym. Chem..

[18] Awaga K., Sugano T., Kinoshita M. (1986). Ferromagnetic intermolecular interactions in a series of organic mixed crystals of galvinoxyl radical and its precursory closed shell compound. J. Chem. Phys..

[19] Nishide H., Yoshioka N., Kaneko T., Tsuchida E. (1990). Poly (p-ethynylphenyl) galvinoxyl: Formation of a new conjugated polyradical with an extraordinarily high spin concentration. Macromolecules.

[20] Nishide H., Hozumi Y., Nii T., Tsuchida E. (1997). Poly (1,2-phenylenevinylene) s bearing nitronyl nitroxide and galvinoxyl at the 4-position: π-conjugated and non-kekule’-type polyradicals with a triplet ground state. Macromolecules.

[21] Wautelet P., Turek P., Le Moigne J. (2002). Synthesis of phenyl ethynylene coupled biradicals and polyradicals based on galvinoxyl. Synthesis.

[22] Miyasaka M., Yamazaki T., Nishide H. (2001). Poly (3-phenylgalvinoxylthiophene). a new conjugated polyradical with high spin concentration. Polym. J..

[23] Nishide H., Kaneko T., Igarashi M., Tsuchida E., Yoshioka N., Lahti P.M. (1994). Magnetic characterization and computational modeling of poly (phenylacetylenes) bearing stable radical groups. Macromolecules.

[24] Kaneko T., Abe H., Namikoshi T., Marwanta E., Teraguchi M., Aoki T. (2009). Synthesis of an optically active poly(aryleneethynylene) bearing galvinoxyl residues and its chiroptical and magnetic properties. Synth. Met..

[25] Kaneko T., Iwamura K., Nishikawa R., Teraguchi M., Aoki T. (2014). Synthesis of sequential poly (1,3-phenyleneethynylene)-based polyradicals and through-space antiferromagnetic interaction of their solid state. Polymer.

[26] Kaneko T., Abe H., Teraguchi M., Aoki T. (2013). Folding-induced through-space magnetic interaction of poly(1,3-phenyleneethynylene)-based polyradicals. Macromolecules.

[27] Anger E., Iida H., Yamaguchi T., Hayashi K., Kumano D., Crassous J., Vanthuyne N., Roussel C., Yashima E. (2014). Synthesis and chiral recognition ability of helical polyacetylenes bearing helicene pendants. Polym. Chem..

[28] Zhao B., Pan K., Deng J.P. (2018). Combining chiral helical polymer with achiral luminophores for generating full-color, on-off, and switchable circularly polarized luminescence. Macromolecules.

[29] Maeda K., Yashima E. (2017). Helical polyacetylenes induced via noncovalent chiral interactions and their applications as chiral materials. Top. Curr. Chem..

[30] Umeda Y., Kaneko T., Teraguchi M., Aoki T. (2005). Helix-sense-selective polymerization of a phenylacetylene bearing an achiral and bulky galvinoxyl moiety. Chem. Lett..

[31] Kaneko T., Umeda Y., Yamamoto T., Teraguchi M., Aoki T. (2005). Assignment of helical sense for poly(phenylacetylene) bearing achiral galvinoxyl chromophore synthesized by helix-sense-selective polymerization. Macromolecules.

[32] Kaneko T., Umeda Y., Jia H.G., Hadano S., Teraguchi M., Aoki T. (2007). Helix-sense tunability induced by achiral diene ligands in the chiral catalytic system for the helix-sense-selective polymerization of achiral and bulky phenylacetylene monomers. Macromolecules.

[33] Katagiri H., Kaneko T., Teraguchi M., Aoki T. (2008). Copper(I) iodide accelerates catalytic activation in rhodium complex-catalyzed helix-sense-selective polymerization of achiral phenylacetylene monomers. Chem. Lett..

[34] Kaneko T., Katagiri H., Umeda Y., Namikoshi T., Marwanta E., Teraguchi M., Aoki T. (2009). Optically active helical structure and magnetic interaction of poly(phenylacetylene)-based polyradicals. Polyhedron.

[35] Klebe J.F., Finkbeiner H., White D.M. (1966). Silylations with bis (trimethylsilyl) acetamide, a highly reactive silyl donor. J. Am. Chem.Soc..

[36] William B.A., Norman B., William J.K., Kreisler S.Y.L. (1981). Facile synthesis of ethynylated benzoic acid derivatives and aromatic compounds via ethynyltrimethylsilane. J. Org. Chem..

