# Supplementary material for: Helix-Sense-Selective Polymerization of 3,5-bis(hydroxymethyl)phenylacetylene Rigidly Bearing Galvinoxyl Residues and Their Chiroptical Properties
Source: Polymers (Basel). 2019 Nov 13;11(11):1877. doi: 10.3390/polym11111877 (PMC6918253; doi:10.3390/polym11111877)
Supplement: Supplementary file 1 [file polymers-11-01877-s001.pdf]

Supporting Information

Helix-Sense-Selective Polymerization of 3,5-Bis(hydroxymethyl)phenylacetylene Rigidly Bearing Galvinoxyl Residues and Their Chiroptical Properties

Zhichun Shi [1,*], Jianjun Wang [4], Masahiro Teraguchi [2,3], Toshiki Aoki [2,3,4] and Takashi Kaneko [2,3,*]

1 College of Chemistry and Chemical Engineering, Qiqihar University, Wenhua Street 42, Qiqihar, Heilongjiang 161006, China; shizhichun1008@163.com (Z.S.)

2 Department of Chemistry and Chemical Engineering, Niigata University, Ikarashi 2-8050, Nishi-ku, Niigata 950-2181, Japan; teraguti@eng.niigata-u.ac.jp (M.T.); toshaoki@eng.niigata-u.ac.jp (T.A.)

3 Graduate School of Science and Technology, Niigata University, Ikarashi 2-8050, Nishi-ku, Niigata 950-2181, Japan

4 College of Materials Science and Engineering, Qiqihar University, Wenhua Street 42, Qiqihar, Heilongjiang 161006, China; wangjianjun860505@163.com

* Correspondence: shizhichun1008@163.com (Z.C.S.); kanetaka@gs.niigata-u.ac.jp (T.K.); Tel & Fax: +81-25-262-6909 (T.K.)

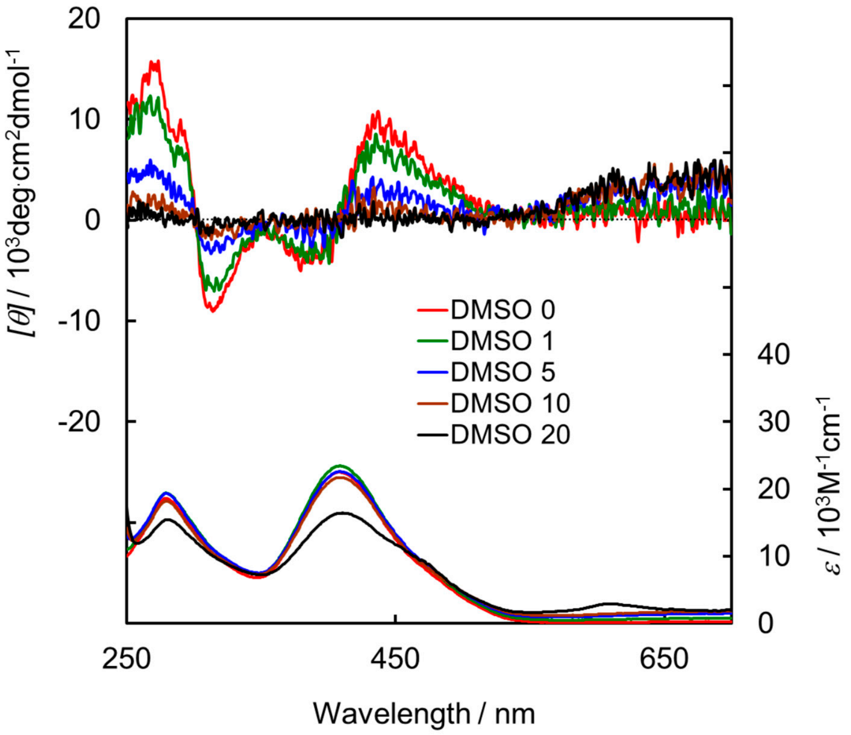

Figure S1. CD and UV-*vis* absorption spectra of poly(*m*-HGDHPA) in THF/DMSO with various compositions.

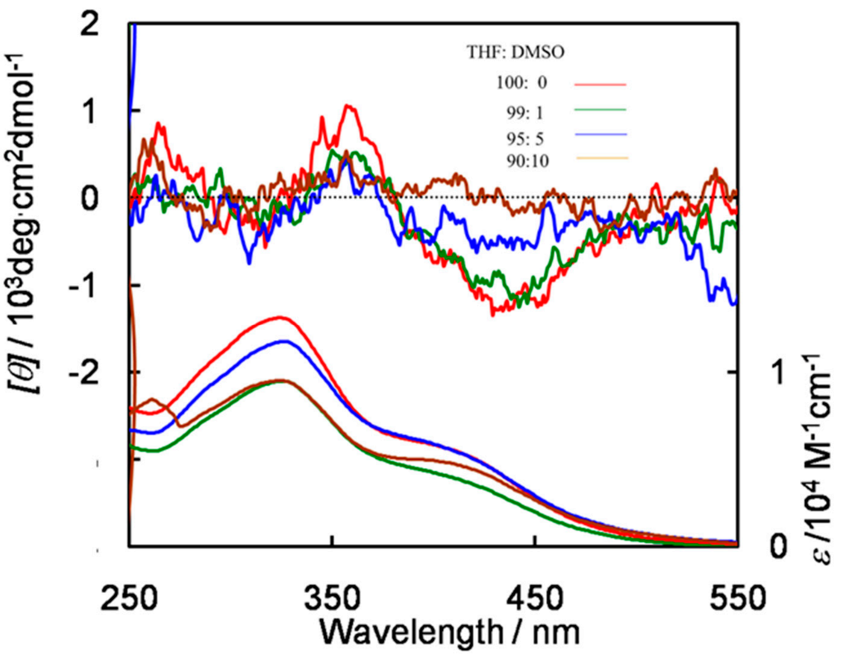

Figure S2. CD and UV-*vis* absorption spectra of poly(*m*-HGTHPA) at 20°C in THF/DMSO.

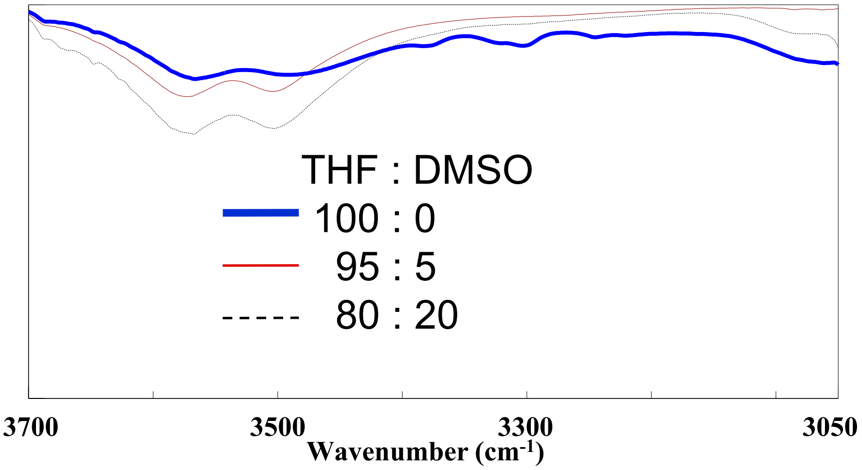

Figure S3. IR spectra of poly(*m*-HGDHPA) in THF/DMSO with various compositions.

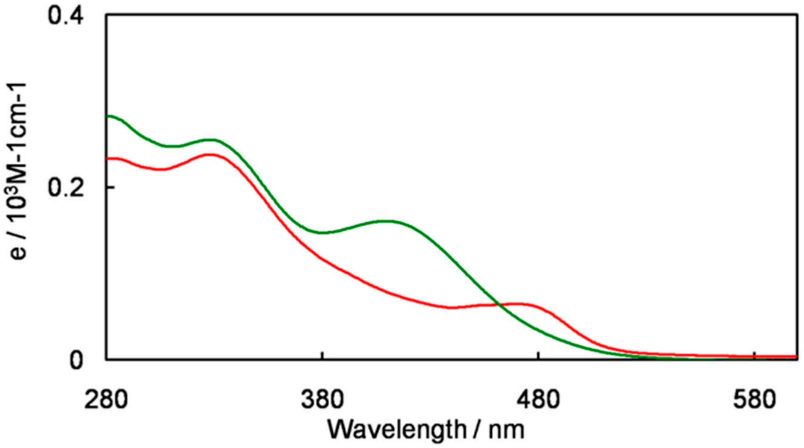

Figure S4. UV-vis absorption spectra of poly(*m*-HGTHPA) (red line: oxidation of poly(*m*-HGTHPA), green line: poly(*m*-HGTHPA)) at 20 °C in THF.

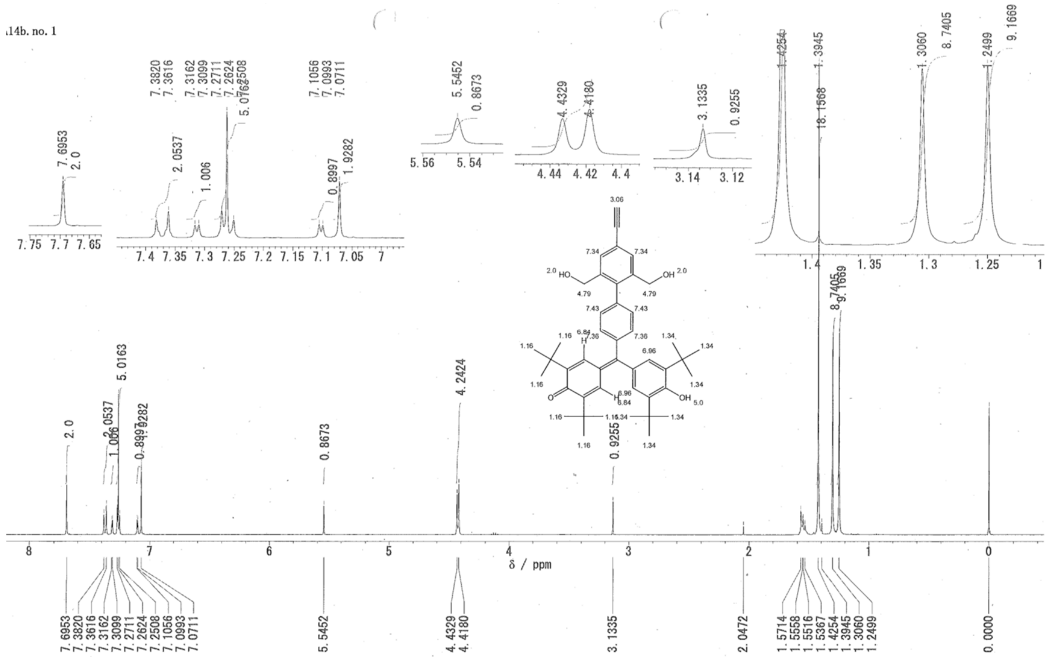

Figure S5. ^{1}H NMR ($CDCl_3$, 400 MHz) spectrum of *p*-HGDHPA.

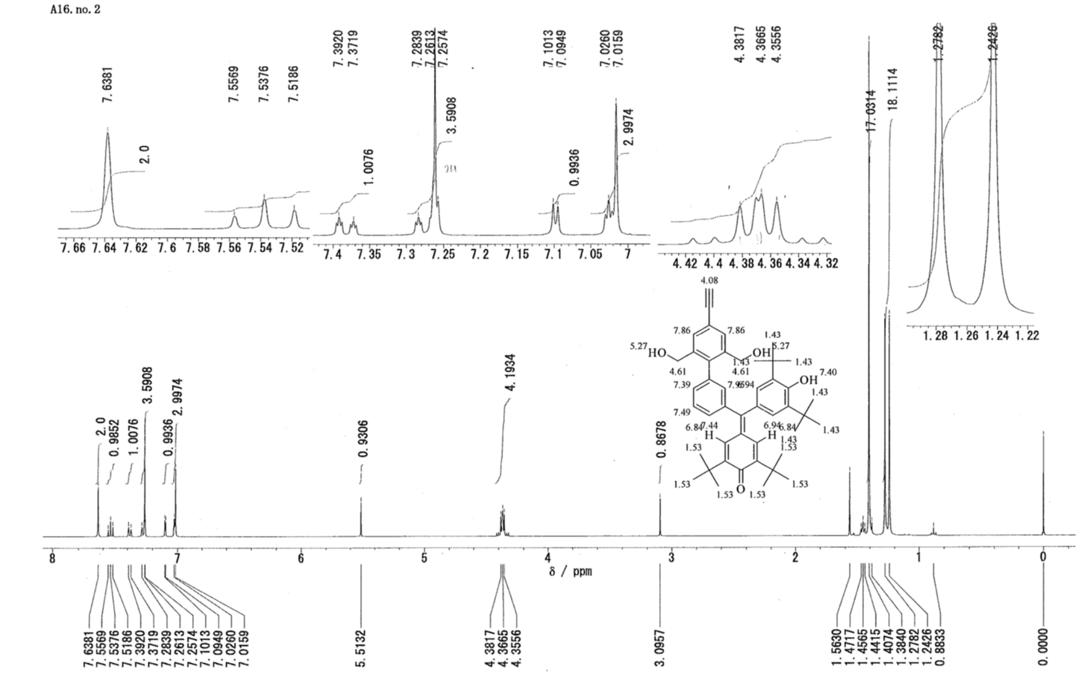

Figure S6. ^{1}H NMR ($CDCl_3$, 400 MHz) spectrum of *m*-HGDHPA.

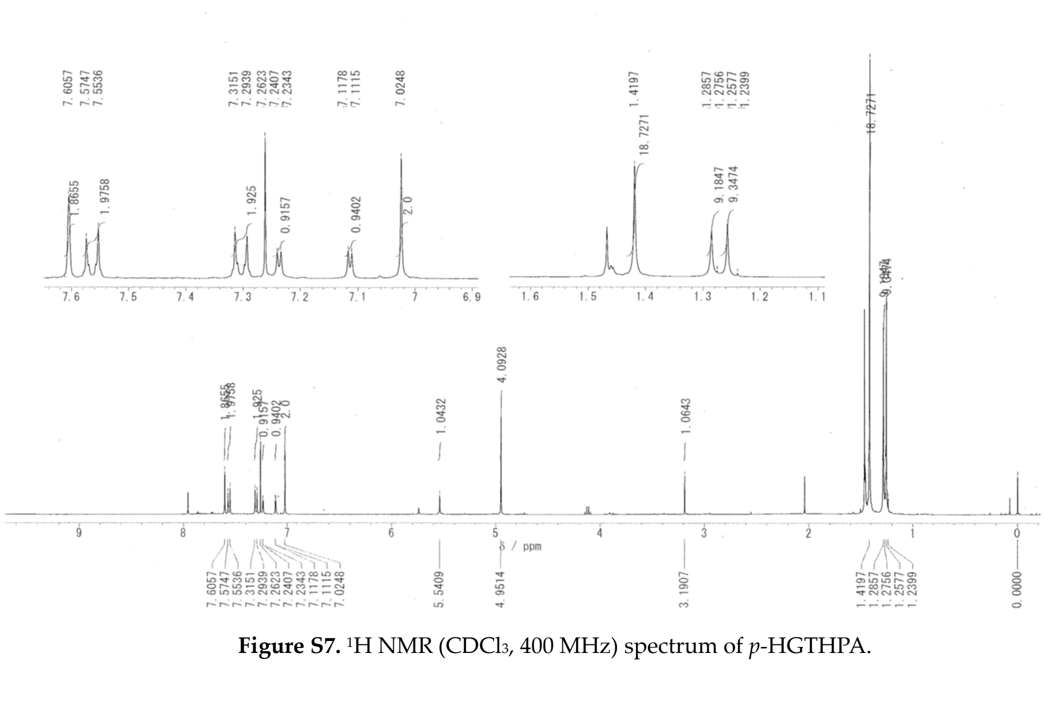

Figure S7. ^{1}H NMR ($CDCl_3$, 400 MHz) spectrum of *p*-HGTHPA.

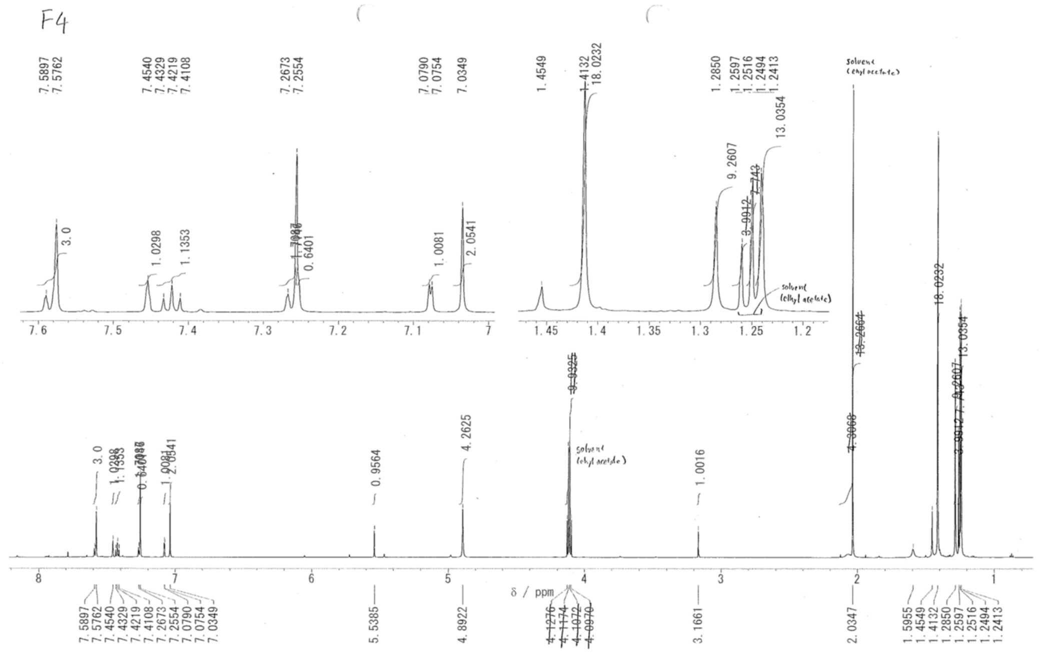

Figure S8. ^{1}H NMR ($CDCl_3$, 700 MHz) spectrum of *m*-HGTHPA.

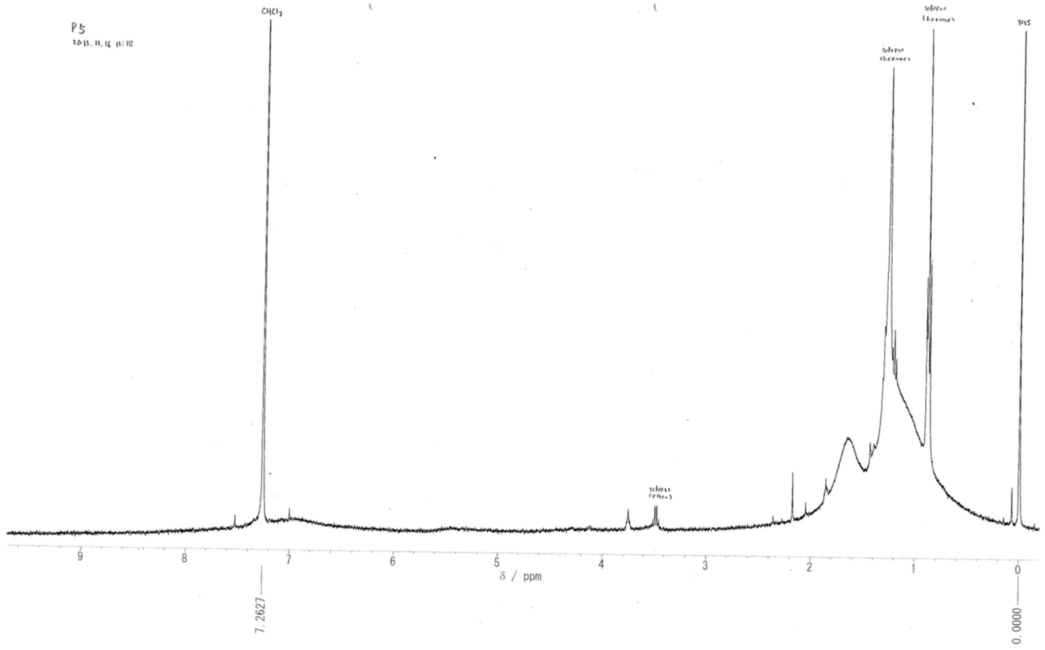

Figure S9. ^{1}H NMR ($CDCl_3$, 400 MHz) spectrum of poly(*m*-HGDHPA).

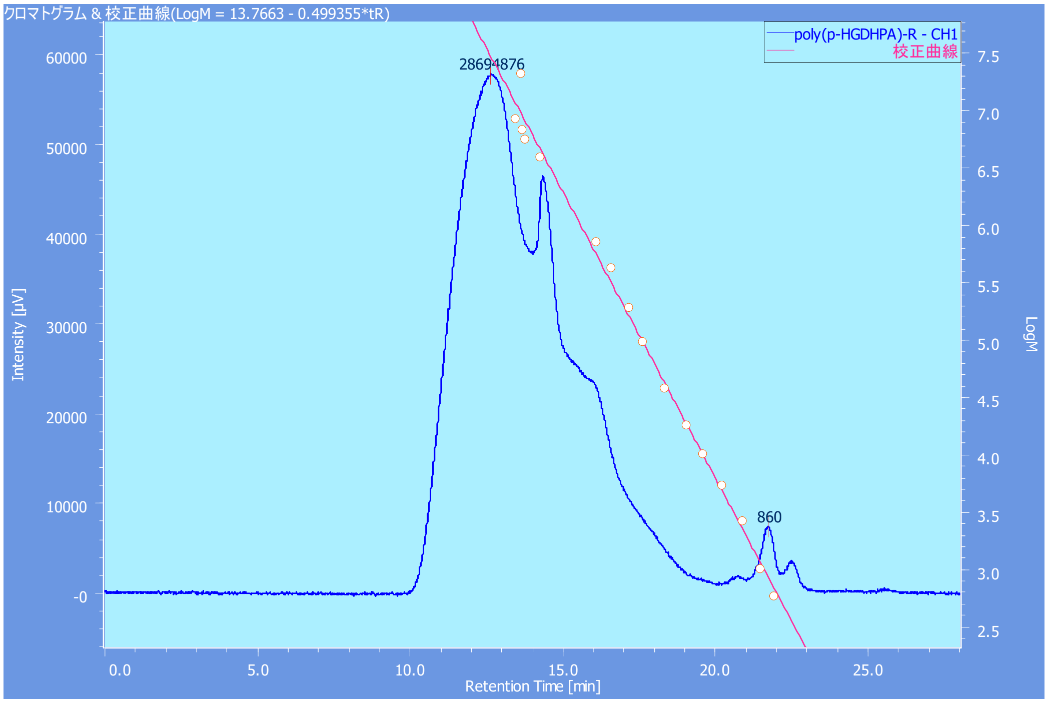

Figure S10. GPC profile of poly(*p*-HGDHPA) in Table 1 no. 1.

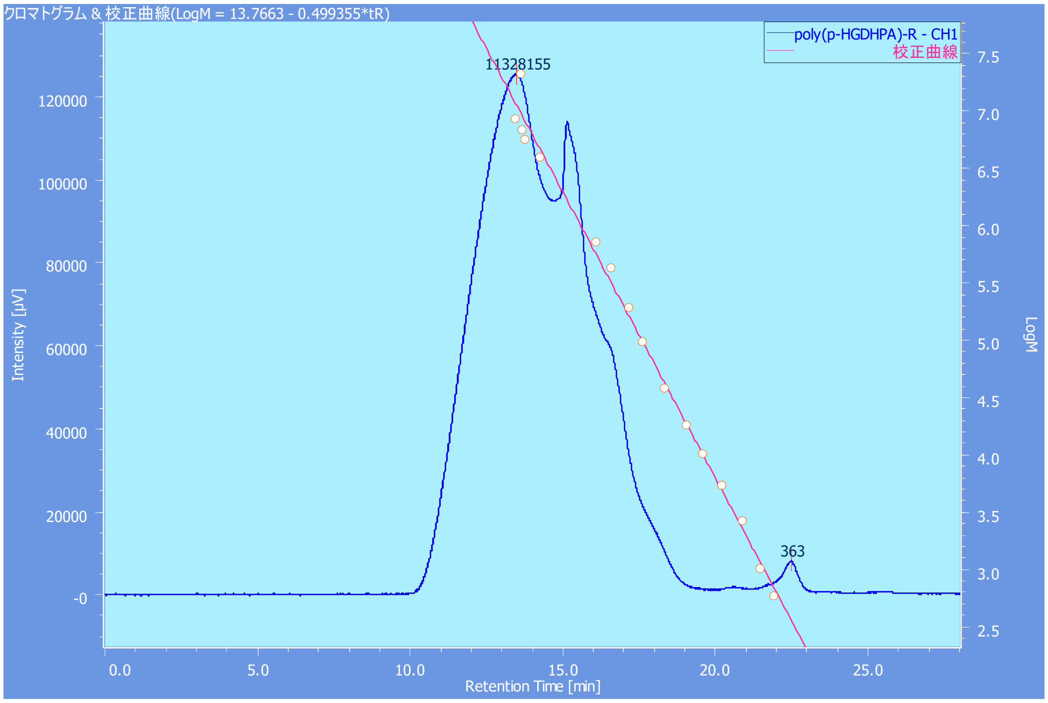

Figure S11. GPC profile of poly(*p*-HGDHPA) in Table 1 no. 2.

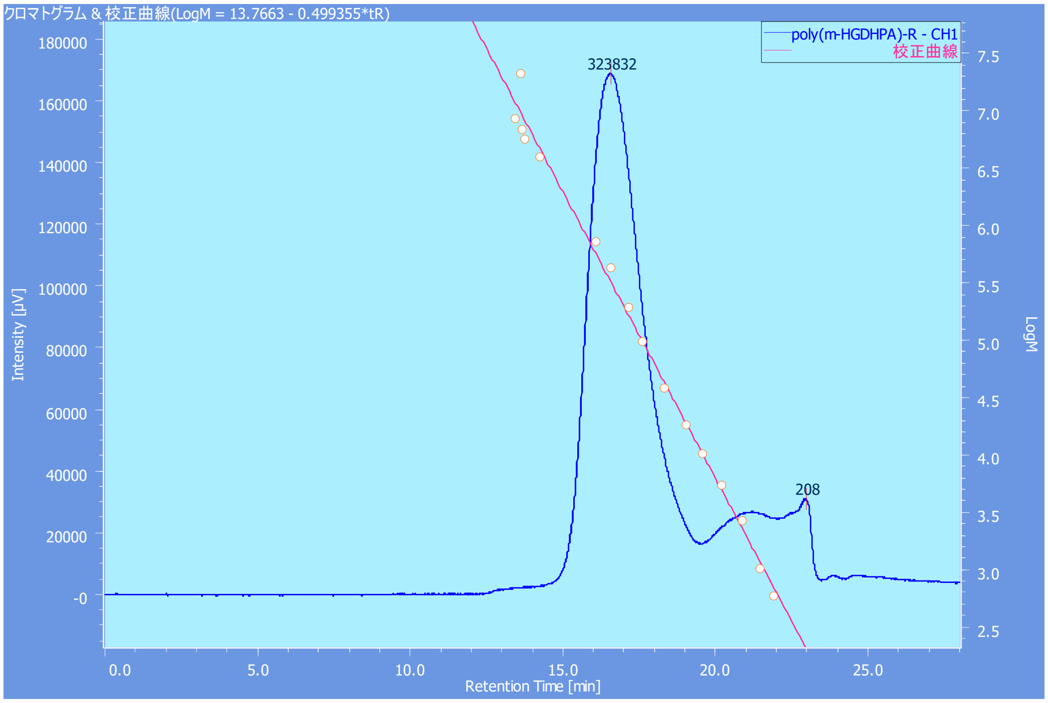

Figure S12. GPC profile of poly(*m*-HGDHPA) in Table 1 no. 3.

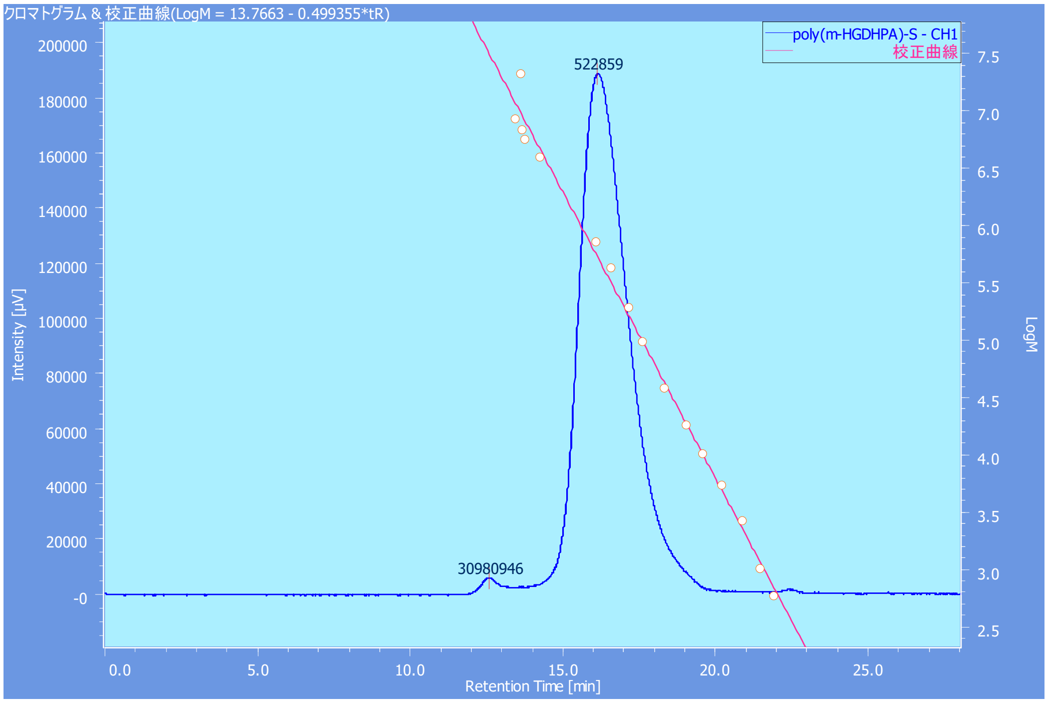

Figure S13. GPC profile of poly(*m*-HGDHPA) in Table 1 no. 4.

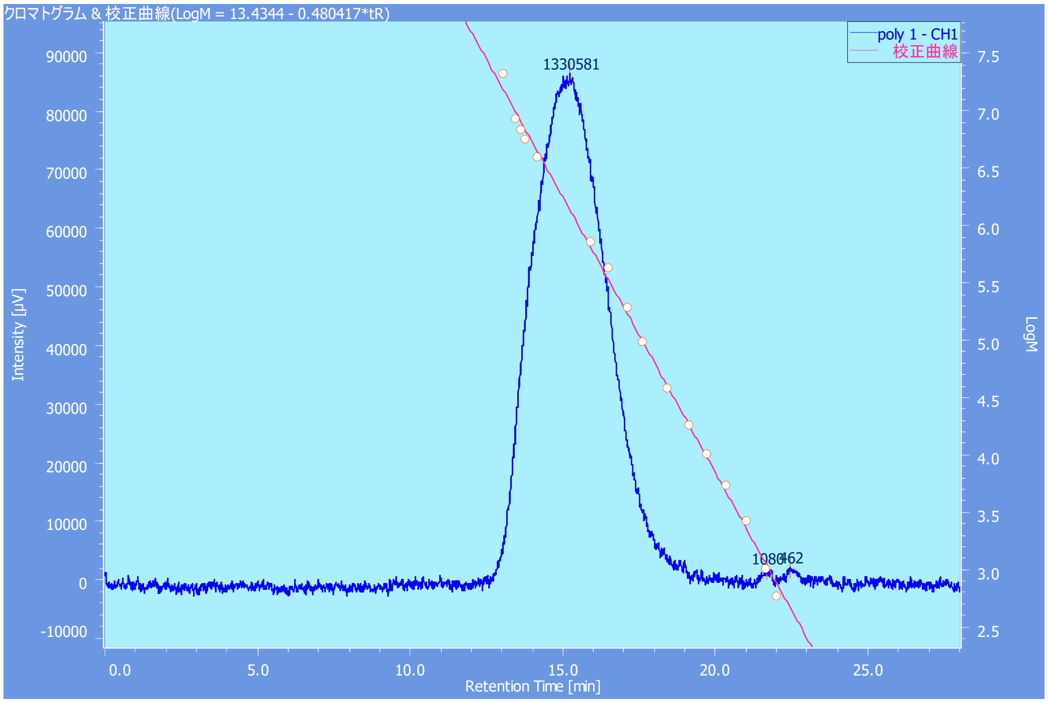

Figure S14. GPC profile of poly(*m*-HGDHPA) in Table 1 no. 5.

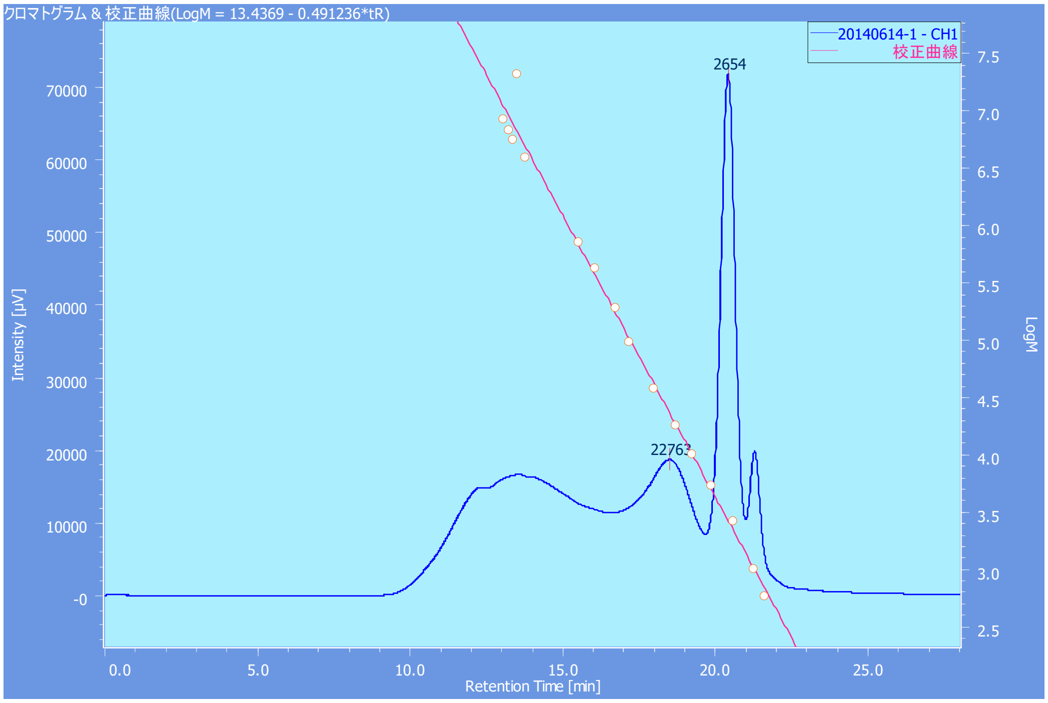

Figure S15. GPC profile of poly(*m*-HGTHPA) in Table 1 no. 6.

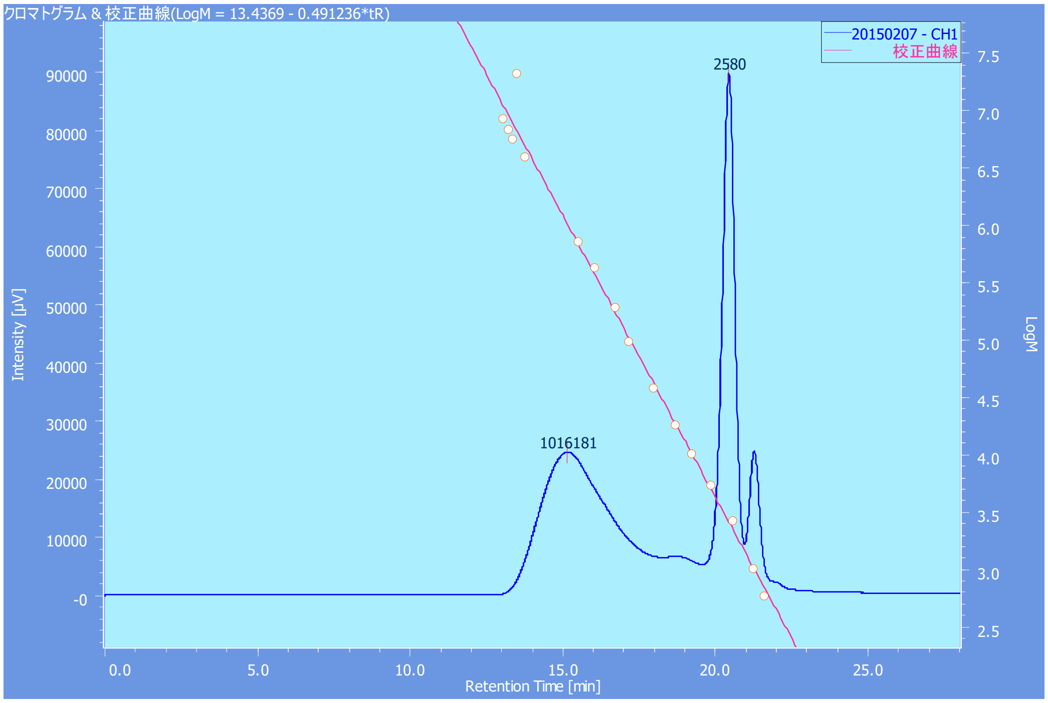

Figure S16. GPC profile of poly(m-HGTHPA) in Table 1 no. 7.
